# Polymerase chain reaction detection of *Leishmania* DNA in skin biopsy samples in Sri Lanka where the causative agent of cutaneous leishmaniasis is *Leishmania donovani*

**DOI:** 10.1590/0074-02760150286

**Published:** 2015-12

**Authors:** Shalindra Ranasinghe, Renu Wickremasinghe, Sanjeeva Hulangamuwa, Ganga Sirimanna, Nandimithra Opathella, Rhaiza DC Maingon, Vishvanath Chandrasekharan

**Affiliations:** 1 University of Sri Jayewardenepura, Faculty of Medical Sciences, Department of Parasitology, Nugegoda,Sri Lanka; 2 Teaching Hospital Anuradhapura, Dermatology Unit, Anuradhapura, Sri Lanka; 3 National Hospital of Sri Lanka, Dermatology Unit, Colombo, Sri Lanka; 4 Divisional Hospital Galenbindunuwewa, Galenbindunuwewa, Sri Lanka; 5 Keele University, Centre for Applied Entomology and Parasitology, Staffordshire, UK; 6 University of Colombo, Faculty of Science, Department of Chemistry, Colombo, Sri Lanka

**Keywords:** cutaneous leishmaniasis, Leishmania donovani, PCR-RFLP, Sri Lanka

## Abstract

*Leishmania donovani* is the known causative agent of both cutaneous (CL) and visceral leishmaniasis in Sri Lanka. CL is considered to be under-reported partly due to relatively poor sensitivity and specificity of microscopic diagnosis. We compared robustness of three previously described polymerase chain reaction (PCR) based methods to detect*Leishmania* DNA in 38 punch biopsy samples from patients presented with suspected lesions in 2010. Both, *Leishmania*genus-specific JW11/JW12 KDNA and LITSR/L5.8S internal transcribed spacer (ITS)1 PCR assays detected 92% (35/38) of the samples whereas a KDNA assay specific for*L. donovani* (LdF/LdR) detected only 71% (27/38) of samples. All positive samples showed a *L. donovani* banding pattern upon HaeIII ITS1 PCR-restriction fragment length polymorphism analysis. PCR assay specificity was evaluated in samples containing *Mycobacterium tuberculosis*, *Mycobacterium leprae,* and human DNA, and there was no cross-amplification in JW11/JW12 and LITSR/L5.8S PCR assays. The LdF/LdR PCR assay did not amplify *M. leprae* or human DNA although 500 bp and 700 bp bands were observed in *M. tuberculosis* samples. In conclusion, it was successfully shown in this study that it is possible to diagnose Sri Lankan CL with high accuracy, to genus and species identification, using *Leishmania* DNA PCR assays.

Leishmaniasis, a neglected tropical disease prevalent in 98 countries (Alvar et al. 2012), has new emerging foci in Sri Lanka (Karunaweera et al. 2003). It has been two decades since the first endemic case of cutaneous leishmaniasis (CL) was reported from Southern Sri Lanka (Athukorale et al. 1992). CL remained sporadic until the year 2000, when more CL cases were reported in soldiers engaged in the jungles in the northern part of the country (Siriwardana et al. 2003, 2010, Karunaweera 2009). In 2008, CL was declared a notifiable disease in the country (MS Sri Lanka 2009). The annual case incidence of CL rose from 674 in December 2009 to 1,365 in December 2014 (MS Sri Lanka 2010,2015a), probably due to more efficient case detection and reporting measures, and increased numbers of infected patients (Alvar et al. 2012). The cumulative CL case incidence from January 2015-March 2015 was 154 (MS Sri Lanka 2015b). Visceral leishmaniasis (VL) could be under reported or under diagnosed in the country since only three confirmed cases of endogenous VL had been reported from Sri Lanka to date (Abeygunasekara et al. 2007, Ranasinghe et al. 2012). Genetically different *Leishmania donovani* zymodeme MON-37 strains have been suggested to cause autochthonous CL and VL in Sri Lanka (Karunaweera et al. 2003, Ranasinghe et al. 2012, Zhang et al. 2014).

In spite of the Public Health importance of CL in Sri Lanka, diagnosis continues to be mostly based on clinical features and traditional diagnostic techniques, i.e., detection of amastigotes in Giemsa stained slit skin smears (SSSs), and punch biopsy and histology (Ranawaka et al. 2012). However, the sensitivity of microscopic SSS and histological diagnosis from Sri Lankan patients was in the 33-45.6% range (Ranawaka et al. 2012), greatly lower than the 60-70% sensitivity range reported for patients from CL foci in other countries (Al-Hucheimi et al. 2009, Chouihi et al. 2009). Furthermore, the reported sensitivity of in vitro cultures of Sri Lankan strains of *L. donovani*was only 40% (Ihalamulla et al. 2008).

Different DNA-polymerase chain reaction (PCR) assays have been described to detect*Leishmania* in clinical samples and to determine the disease causative species (El Tai et al. 2000, Kuhls et al. 2005, Mosleh et al. 2015, Rodríguez-Brito et al. 2015). KDNA PCR has shown to have > 90% sensitivity and 100% specificity in detecting *Leishmania* DNA in clinical samples (Salotra et al. 2001, Nasreen et al. 2012). Internal transcribed spacer (ITS)1 PCR followed by restriction fragment length polymorphism (RFLP) analysis has also been widely used to identify*Leishmania* parasite to species and subspecies level (Akhoundi et al. 2013, Hajjaran et al. 2013, Mosleh et al. 2015).

A spectrum of skin lesions have been reported in Sri Lankan CL patients ranging from papules, nodules, dry, and wet ulcers, to plaques (Ranawaka et al. 2012). This observed clinical polymorphism raises the need to re-examine parasite contribution to clinical outcome and the existence of more*Leishmania* species in addition to *L. donovani* in Sri Lanka. Furthermore, differential diagnosis of infective causes of chronic granulomatous skin lesions in Sri Lanka implies distinguishing between CL, lupus vulgaris, and scrofuloderma [cutaneous tuberculosis (TB)] and leprosy. However, no systematic evaluation of DNA based diagnostic tools to detect*Leishmania* DNA in cutaneous lesions in Sri Lanka has been reported to date. In this study, three different *Leishmania* DNA PCR assays were compared regarding sensitivity and specificity for identifying*Leishmania* to the species level in 38 skin biopsy samples from patients from an endemic CL focus in the north-central province of Sri Lanka where cutaneous TB and leprosy are also endemic (Ranawaka et al. 2010, Rathnayake et al. 2010,Wijesinghe & Wijesinghe 2013, MS Sri Lanka 2014).

## SUBJECTS, MATERIALS AND METHODS

*Patients and ethics -* Clinically suspected CL cases living or working in the north-central province of Sri Lanka who were passively reported to the dermatology clinics of the Teaching Hospital Anuradhapura and the National Hospital of Sri Lanka between January-June 2010 were included in the study. Clinical diagnosis was made by the consultant dermatologists as described (Siriwardana et al. 2003, Bari 2012, Ranawaka et al. 2012). Ethical approval was obtained from the Ethical Review Committee (ERC), Faculty of Medical Sciences, University of Sri Jayewardenepura, Sri Lanka. Written informed consent was obtained from every literate adult participant and from either of the parents or the guardian before including a child into the study. Since informed consent was oral for illiterate participants their ERC approved thumbprint consent was used in this study. All information collected was kept under confidential cover. Any patient having foreign exposure to a leishmaniasis endemic country during any time of their life and/or who did not give written informed consent was excluded from the study.

*Sample size calculation -* Three pre-described*Leishmania* DNA PCR assays were selected and tested for their robustness in diagnosing CL of Sri Lankan origin (Rodgers et al. 1990, El Tai et al. 2000, Salotra et al. 2001). The sample size was calculated as follows: the standard normal deviation for two-sided α with a 95% confidence level was 1.96, the estimated proportion (sensitivity) was 0.9 based on the published data (Salotra et al. 2001), and the width of confidence interval was 0.2. The calculated sample size was n = 35 (Hulley et al. 2001). In this study, n = 38 samples from 38 patients were used.

*SSS and punch biopsy -* SSSs from 38 clinically suspected previously undiagnosed and untreated CL lesions were obtained by a clinician. The slides were stained with Giemsa and examined under a light microscope under oil immersion in the Department of Parasitology, Faculty of Medical Sciences, University of Sri Jayewardenepura. Amastigotes were identified as having round to ovoid shape and characterised by a distinctive nucleus and adjacent kinetoplast. A diameter of 2-4 mm punch biopsy samples from the active edge of the same suspected lesion, taken by a clinician under local anaesthesia and sterile conditions, were stored in Net buffer [150 mM NaCl, 15 mM Tris-HCL (pH 8.3), 1 mM ethylenediamine tetraacetic acid (EDTA)] prior to DNA extraction (Salotra et al. 2001). These punch biopsy samples were initially stored at -40°C at the Teaching Hospital Anuradhapura, transported on ice to the Department of Parasitology, Faculty of Medical Sciences, University of Sri Jayewardenepura, and stored at -80°C until DNA was extracted. Samples collected from the National Hospital of Sri Lanka were brought in Net buffer on ice and frozen at -80°C as described above.

*DNA extraction from positive control culture and skin biopsy samples -* An endogenous in vitro cultured CL causing strain from one of the patients was identified as *L. donovani* MON-37 by Sanger partial DNA sequencing of the 6-phosphogluconate dehydrogenase gene at the McGill University Genome Quebec Innovation Centre, Canada (McCall et al. 2013). This same strain was later confirmed by whole genome sequencing in a different study (Zhang et al. 2014). DNA from this pure culture (at a parasite count of 1 x 10^6^/mL) was extracted using QIAGEN DNeasy blood and tissue kits according to manufacturers’ guidelines, quantified by spectrophotometry (Thermo Fisher Scientific, USA), and used as a positive control. Two millimetre diameter samples from the punch biopsy specimens obtained from SSS positive CL patients were cut on a sterile glass slide using a sterile scalpel blade and DNA was extracted using the same DNA extraction kit.

*PCR -* Previously described JW11/12 primer set amplifies a 120 bp fragment of KDNA of genus *Leishmani*a (Rodgers et al. 1990), the LdF/LdR primer set amplifies a 600 bp fragment in *L. donovani* species-specific KDNA (Salotra et al. 2001), and the LITSR/L5.8S set amplifies a 320 bp fragment of ITS1 region of *Leishmania*genus-specific DNA (El Tai et al. 2000). SSS positivity was taken as the gold standard. For each PCR assay, any SSS positive sample that became negative by first PCR was repeated three times on three consecutive days. Similarly, any sample that became positive by one set of primers and negative with a different set of primers was subjected to three repeat PCRs to conclude a negative PCR result given by the specific primer pair. Sensitivity and specificity assays were performed with all three sets of primers.

*PCR conditions -* A volume of 2 µL of extracted DNA from punch biopsy samples or from pure *L. donovani* culture was amplified with 100 pmol of each forward and reverse primers in the presence of 1.5 mM MgCl_2_, 25 mM Tris-HCL (pH 9.0), 25 mM NaCl, 200 µM each deoxynucleotide triphosphate, 50 units/mL *Taq* DNA polymerase (*Thermus acquaticus*) (PCR master mix; Promega, USA) in a final volume of 10 µL. Primer sequences and relevant PCR conditions are described in the Table. A volume of 5 µL from the PCR products were run on 1-1.75% (w/v) wide range agarose gel stained with 0.2 µg/mL ethidium bromide (EtBr) (Sigma Aldrich) in 1X tris-acetate-EDTA buffer at 100 V for 45 min, visualised under ultraviolet light and images captured by a computerised gel documentation unit (Quantum ST5; Vilber Lourmat, Germany).

**Table:** TABLE. Polymerase chain reaction conditions

PCR	Primer sequence	Amplification programme
KDNA genus *Leishmania* specific PCR (Rodgers et al. 1990)	JW11 (forward): 5’-CCTATTTTACACCAACCCCCAGT-3’ JW12 (reverse): 5’-GGGTAGGGGCGTTCTGCGAAA-3’	Initial denaturation at 94°C for 1 min followed by 34 cycles of denaturation at 94°C for 30 s, annealing at 58°C for 30 s, and extension at 72°C for 30 s
KDNA *Leishmania donovani* species-specific PCR (Salotra et al. 2001)	LdF (forward): 5’-AAATCGGCTCCGAGGCGGGAAAC-3’ LdR (reverse): 5’-GGTACACTCTATCAGTAGCAC-3’	Initial denaturation at 94°C for 2 min followed by 40 cycles of denaturation at 94°C for 1 min, annealing at 45°C for 1 min, and extension at 72°C for 2 min, with a final extension at 72°C for 3 min
Internal transcribed spacer 1 PCR genus*Leishmania* specific (El Tai et al. 2000)	LITSR (forward): 5’-CTGGATCATTTTCCGATG-3’ L 5.8S (reverse): 5’-TGATACCACTTATCGCACTT-3’	Initial denaturation at 95°C for 2 min, followed by 34 cycles of denaturation at 95°C for 20 s, annealing at 53°C for 30 s, and extension at 72°C for 1 min, with a final extension of 72°C for 6 min

*Sensitivity assays -* Sensitivity assays were performed by carrying out PCR with the three selected sets of primers in the presence of 10-fold serial dilutions, ranging from 100 ng to 1 fg of CL in vitro culture *L. donovani* DNA prepared in molecular biology grade water.

*Negative controls and specificity assays -* Each endpoint PCR reaction contained a PCR reaction with no DNA. The selected*Leishmania* primers were tested for cross amplification with five samples of each *Mycobacterium tuberculosis* and*Mycobacterium leprae,* and human DNA. The *M. leprae* samples were extracted from smear positive leprosy skin biopsy samples and were tested with previously described *M. leprae-*specific primers for the presence of *M. leprae* DNA (Banerjee et al. 2008). Pre-tested DNA samples extracted from five different *M. tuberculosis* in vitro cultures were kindly supplied by Prof Jennifer Perera, Department of Microbiology, Faculty of Medicine, University of Colombo, Sri Lanka. All PCR positive samples, i.e., the samples that were positive with *Leishmania* genus-specific primers (JW11/12 & LITSR/L5.8S) (n = 35) and negative with *L. donovani-*specific primers (LdF/LdR) (n = 8), were subjected to ITS1 PCR (LITSR/L5.8S), *Hae*III digestion, and RFLP analysis. The restriction sites for *Hae*III were obtained from RestrictionMapper v.3 (restrictionmapper.org/). A 10 µL volume of the selected ITS1 PCR products was digested with 10 U of *Hae*III at 37°C for 2 h. A volume of 10 µL of the digested products was then run for 45 min at 100 V on a wide range 1.75% (w/v) agarose gel (Invitrogen) in 0.5 x Tris-boric acid-EDTA followed by staining with 0.2 µg/mL EtBr (Sigma Aldrich). The gel images were captured as described above.

## RESULTS

**Fig. 1:**
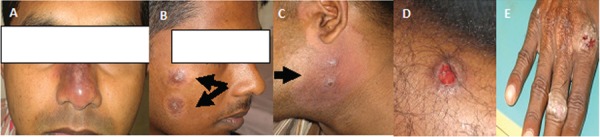
: spectrum of clinical presentations observed in cutaneous leishmaniasis study patients from Sri Lanka. A: plaque like lesion; B: nodules with a depigmented halo (arrow); C: two volcano-like lesion with depigmentation (arrow); D: wet ulcer; E: dry scaly lesion. All patients presented from the north-central province of Sri Lanka.

*Spectrum of clinical presentations -* The spectrum of clinical presentations in the collected CL samples varied from papules, nodules, noduloulcerative (volcano-type) lesions, dry ulcers, wet ulcers to scaly plaques. These lesions were located in exposed areas of the body. A number of lesions had a depigmented halo (Fig. 1).

**Fig. 2:**
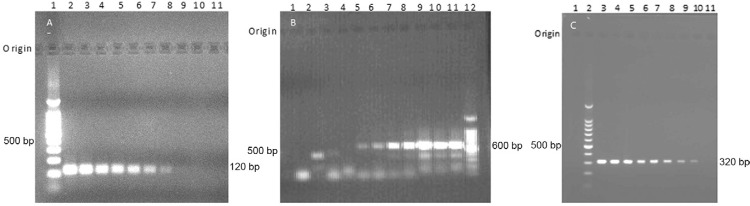
: sensitivity of different polymerase chain reaction (PCR) primer assays. A-C: 10-fold DNA serial dilutions from *a Leishmania donovani* pure culture were used in PCR reactions with different primer sets as indicated; A: JW 11/12 KDNA PCR (Rodgers et al. 1990) [Lane 1: 100 bp DNA ladder; L2-10: 10-fold descending serial dilutions of *Leishmania* DNA from 100 ng-1 fg; 11: -ve control (no DNA)]; B: LdF/LdR KDNA PCR (Salotra et al. 2001) [Lane 1: -ve control (no DNA); 2-11: 10-fold ascending serial dilutions of*Leishmania* DNA from 0.1 fg-100 ng; 12: 100 bp DNA ladder]; C: LITSR/L5.8S internal transcribed spacer 1 PCR (El Tai et al. 2000) [Lane 1: -ve control (no DNA); 2: 100 bp DNA ladder; 3-11: 10-fold descending serial dilutions of *Leishmania* DNA from 100 ng-1 fg].

*Sensitivity assays -* Genus *Leishmania-*specific PCR with KDNA JW11/12 primers (Fig. 2A) and*L. donovani* species-specific LdF/LdR primers (Fig. 2B) detected as little as 100 fg (1 parasite) *Leishmania* DNA in the positive control *L. donovani* DNA serial dilutions. ITS1 PCR (LITSR/L5.8S) primers detected as little as 10 fg (0.1 parasite) in the serial dilutions (Fig. 2C).

**Fig. 3:**
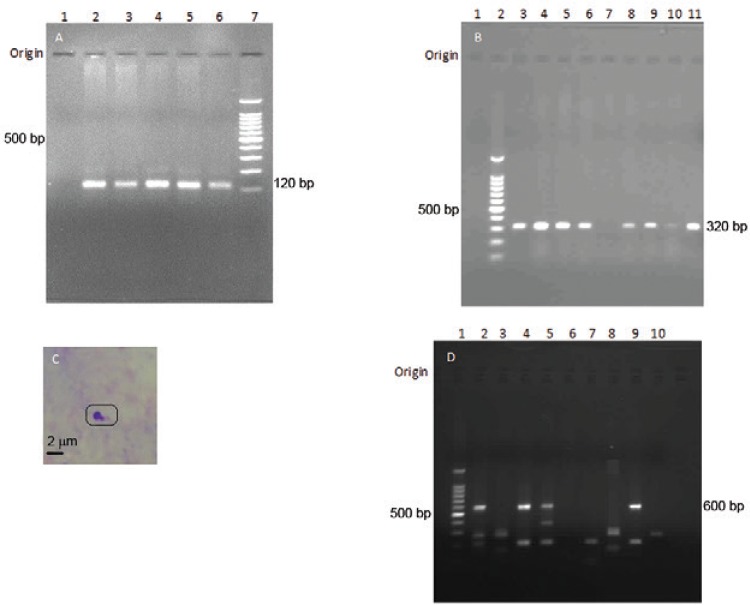
: detection of *Leishmania* DNA in punch biopsy samples with selected primers. A: JW11/12 KDNA polymerase chain reaction (PCR) (n = 4/38 samples) [Lane 1: -ve control (no DNA); 2: cutaneous leishmaniasis (CL) sample 3; 3: CL sample 4; 4: CL sample 10; 5: CL sample 11; 6: +ve control (CL culture); 7: 100 bp DNA ladder]; B: LITSR/L5.8S internal transcribed spacer 1 PCR (n = 8/38 samples) [Lane 1:-ve control (no DNA); 2: 100 bp DNA ladder; 3: CL sample 1; 4: CL sample 3; 5: CL sample 5; 6: CL sample 10; 7: CL sample 12; 8: CL sample 13; 9: CL sample 16; 10: CL sample 23; 11: +ve control (CL culture)]; C: PCR negative Giemsa-stained slit skin smear showing rounded amastigote-like forms; D: LdF/LdR KDNA PCR (n = 8/38 samples) [Lane 1: 100 bp DNA ladder; 2: CL sample 1; 3: CL sample 2; 4: CL sample 3; 5: CL sample 4; 6: CL sample 5; 7: CL sample 7; 8: CL sample 8; 9: +ve control (CL culture); 10: -ve control (no DNA)].

Thirty-five from the 38 study samples became positive with both JW11/12 KDNA primers and ITS1 (LITSR/L5.8S) primers yielding similar sensitivity of 92.1% for pairs of both primers (Fig. 3A, B). Occasional samples that showed negative PCR in the first experiment became positive with a repeat PCR (sample 12, Lane 7 in Fig. 3B). However, three PCR negative samples showed amastigote-like forms with a nucleus and a kinetoplast like morphology and absent cytoplasm in the SSS. The possible parasitological grading was 2+ in all three PCR negative samples (Fig. 3C). These three negative samples were tested for the presence of DNA by spotting 2 µL of each sample with ~250 ng (2 µL) of quantified CL DNA as a positive control on a 1% thin agarose gel. The results showed the presence of DNA in the three PCR negative samples (results not shown). Furthermore, these PCR-negative samples (n = 3) did not show any PCR inhibition when tested by spiking with 1 pg of *L. donovani* DNA from pure cultures and amplified with primers described above (results not shown).

Out of the 35 samples that were tested positive with JW11/12 and LITSR/L5.8S primers, eight samples became negative with LdF/LdR primers yielding a sensitivity of only 71.1% (27/38) (Fig. 3D).

**Fig. 4:**
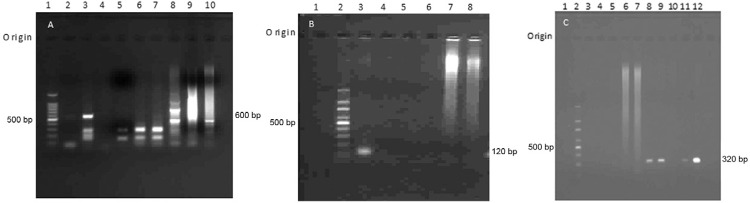
: specificity assays of polymerase chain reaction (PCR). A:*Leishmania* LdF/LdR KDNA PCR in the presence of*Mycobacterium tuberculosis* DNA [Lane 1: 100 bp DNA ladder; 2: cutaneous leishmaniasis (CL) sample 35; 3:*Leishmania* +ve control (CL culture); 4: -ve control (no DNA); 5: CL sample 34; 6: CL sample 37; 7: CL sample 36; 8-10:*M. tuberculosis* samples (n = 3/5)]; B:*Leishmania* JW11/12 KDNA PCR in the presence of human, *Mycobacterium leprae* and *M. tuberculosis* DNA [Lane 1: -ve control (no DNA); 2: 100 bp DNA ladder; 3: +ve control (CL culture); 4-6: *M. leprae*and human DNA samples (n = 3/5); 7-8: *M. tuberculosis*DNA samples (n = 2/5)]; C: LITSR/L5.8S internal transcribed spacer 1 PCR in the presence of human, *M. leprae* and *M. tuberculosis* DNA [Lane 1: -ve control (no DNA); 2: 100 bp DNA ladder; 3-5: *M. leprae* and human DNA samples (n = 3/5); 6-7: *M. tuberculosis* DNA samples (n = 2/5); 8: CL sample 32 (repeat); 9: CL sample 36; 10: CL sample 37 (repeat); 11: CL sample 35 (repeat); 12: +ve control (CL culture)].

*Specificity assays -* LdF/LdR primer assays [reportedly *L. donovani*-specific, Salotra et al. (2001)] amplified 500 bp and 700 bp bands in four and two out of five tested *M. tuberculosis* DNA samples, respectively (Fig. 4A). However, these primers did not show any cross amplification in the presence of *M. leprae* or human DNA (results not shown). Specificity assays in the presence of and *M. tuberculosis*, *M. leprae* and human DNA showed that there was no cross-amplification in *Leishmania* KDNA JW11/12 (Fig. 4B) and LITSR/L5.8S ITS1 primer assays (Fig. 4C).

**Fig. 5:**
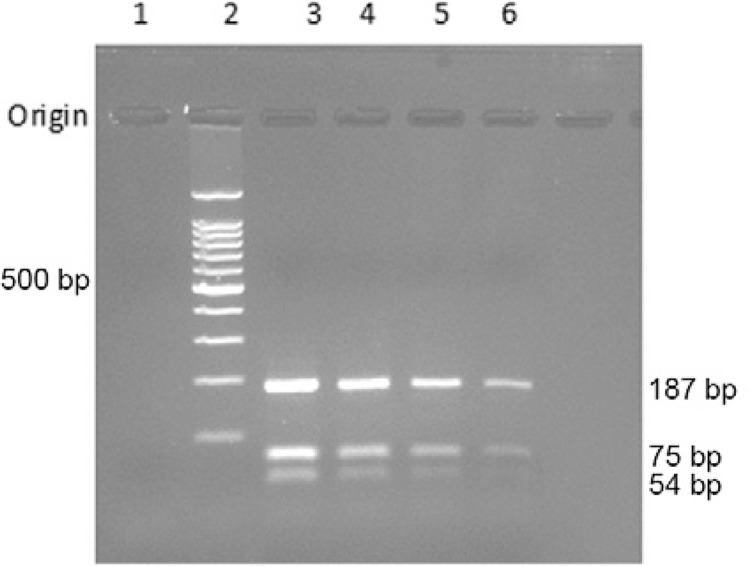
: *Leishmaniadonovani* species-specific restriction fragment length polymorphism- polymerase chain reaction (PCR) assay. Internal transcribed spacer 1 amplified DNA digested with*Hae*III {Lane 1: -ve control [no DNA PCR]; 2: 100 bp DNA ladder; 3: +ve control [cutaneous leishmaniasis (CL) culture]; 4: CL sample 1; 5: CL sample 5; 6: CL sample 10}. Samples 5 and 10 were two out of eight samples which were negative using LdF/LdR primers.

ITS1 PCR *Hae*III RFLP analysis was used to increase species-specificity of the highly sensitive ITS1 PCR assay (Figs 2C, 5). The calculated sizes of the expected fragments after digestion with*Hae*III were 54, 75, and 187 bp according to the *L. donovani* ITS1 sequence (GenBank accession AM901448.1). All 35 samples including the eight LdF/LdR PCR negative samples gave a similar *L. donovani* banding pattern upon ITS1 PCR *Hae*III RFLP (Fig. 5).

## DISCUSSION

This study for the first time systematically evaluated DNA based methods to detect*Leishmania* DNA in skin biopsy samples taken from Sri Lankan CL lesions. Out of the three sets of primers tested both the JW11/12 KDNA (Rodgers et al. 1990) and the LITSR/L5.8S ITS1 primer pairs (El Tai et al. 2000) showed high sensitivity (92%) with 100% specificity when compared to 71% sensitivity shown by the third LdF/LdR primer set (Salotra et al. 2001) (Fig. 4). Although a clinical polymorphism was detected in the CL presentations in Sri Lanka, the *L. donovani* specific LdF/LdR PCR and the ITS1 PCR- RFLP revealed that the only causative organism in all 35 PCR positive tested samples was *L. donovani*. Although parasite virulence factors might account for strain or clone-specific clinical presentations (WHO 2010, McCall & McKerrow 2014), it has been also shown that different host cytokines, chemokines may contribute to different clinical outcomes in leishmaniasis (Carvalho et al. 2012, Hartley et al. 2012).

The specificity assays carried out in this study revealed unexpected 500 bp and 700 bp bands in four and two out of five tested *M. tuberculosis* DNA samples, isolated from five different cultures when amplified with *L. donovani*-specific LdF/LdR primers. Thus, the use of LdF/LdR primers would produce complex diagnostic banding patterns in geographic regions including Sri Lanka where cutaneous TB is one of the differential diagnoses of infective causes of chronic skin lesions with leishmaniasis. Salotra et al. (2001) reported no LdF/LdR primers cross-amplification with *M. tuberculosis* DNA using blood samples from patients with pulmonary TB. The reported sensitivity of PCR in detecting *M. tuberculosis* DNA in blood of pulmonary and extra-pulmonary TB patients was low (< 50%) (Crump et al. 2012, Kashyap et al. 2013). Therefore, the negative results reported could have been most likely due to false negatives or absence of*M. tuberculosis* DNA in the tested blood samples (true negatives). It was also observed that LdF/LdR primers give bands in 400 bp and 300 bp region in some CL positive samples in addition to the 600 bp region (Lane 5 inFig. 3D, Lanes 3, 5-7 in Fig. 4A). This could be due to intraspecies polymorphism in the KDNA as described by Srivastava et al. (2011), and the presence of mini-circle subclasses, and sequence heterogeneity (Ceccarelli et al. 2014).

However, it should be emphasised that in the present study LdF/LdR primers did not yield a *L. donovani* specific 600 bp amplicon in any of the negative controls including *M. tuberculosis*, *M. leprae* or human DNA. Thus, *L. donovani-*specific LdF/LdR primers (Salotra et al. 2001) could be used successfully to detect *L. donovani* DNA in skin biopsy samples in low income setups.

Although ITS1 PCR-*Hae*III RFLP analysis appeared to be the best molecular diagnostic tool evaluated in this study to detect *L. donovani* DNA in skin biopsy samples, it will be costlier than end point LdF/LdR PCR. When the diagnostic purpose is to detect *Leishmania*genus-specific DNA in skin biopsy samples, either ITS1 PCR with LITSR/L5.8S primers or *Leishmania* KDNA primers JW11/12 would be successful due to a 92% sensitivity and 100% specificity shown in the study. Our study emphasises the importance of laboratory-based in addition to clinical diagnosis of CL as suggested by Siriwardana et al. (2015). An interesting conclusion, to be confirmed by larger sample size studies, is that *L. donovani* is the only species causing polymorphic clinical presentations in Sri Lanka. However, the relative contributions of strain polymorphism and host-related immune factors to the observed clinical spectrum deserve further investigation.

It is important to note that leishmaniasis is a spreading disease in the country warranting the implementation of active case detection, early treatment and integrated vector control at national level. In this context, 1% SSS positive newly diagnosed CL case incidence was reported in a cross sectional study in a high CL endemic area in the north-central province of Sri Lanka (Ranasinghe et al. 2013), indicating the need for active case detection measures. A recent study described the successful application of a PCR method along with SSS and culture for active case detection in another CL endemic area in southern Sri Lanka (Kariyawasam et al. 2015). Furthermore, limited data on epidemiology show evidence of geographical overlap of cutaneous TB, leprosy, and CL in Sri Lanka (Ranawaka et al. 2010, Yasaratne & Madegedara 2010, MS Sri Lanka 2014, 2015a). Coinfections of different clinical forms of TB and leprosy with leishmaniasis have also been reported in endemic geographic regions including Sri Lanka (Delobel et al. 2003, Rathnayake et al. 2010). Therefore, these highly sensitive and specific ITS1 (LITSR/L5.8S) and JW11/12 PCR methods evaluated with local samples in this study would be valuable and cost-effective tools for active case detection of clinically suspected CL patients with negative SSS and culture and for epidemiological studies even in areas where skin TB and leprosy coexist with leishmaniasis.
